# Population-Based Quantitative Spatial Mapping of Ulnar Motor Branch Origins in the Forearm: An Integrated Anatomical Reference Framework

**DOI:** 10.3390/diagnostics16172830

**Published:** 2026-09-02

**Authors:** Alexandre Merí-Vived, Pedro Víctor López Plaza, Antoni Morral Fernández, Manuel Llusà-Pérez, Marcel García-Cuesta, Aroa Casado Rodríguez

**Affiliations:** 1 School of Health Science Blanquerna, Universitat Ramon Llull, 08025 Barcelona, Spain; pedrovictorlp@blanquerna.url.edu (P.V.L.P.); antonimf@blanquerna.url.edu (A.M.F.); 2 Unit of Human Anatomy and Embryology, Department of Surgery and Medical-Surgical Specialties, Faculty of Medicine and Health Sciences, University of Barcelona (Campus Clínic), 08036 Barcelona, Spain; mllusa@ub.edu (M.L.-P.); m.garciacuesta@ub.edu (M.G.-C.); 3 Faculty of Medicine, University of Vic–Central University of Catalonia, 08500 Vic, Spain; 4 Department of Medicine and Life Sciences, Pompeu Fabra University, 08003 Barcelona, Spain

**Keywords:** ulnar nerve, motor branch origins, flexor carpi ulnaris, flexor digitorum profundus, quantitative anatomy, anthropometric normalization, musculotendinous landmarks, spatial mapping, cadaveric study

## Abstract

**Background/Objectives**: The extramuscular branching pattern of the ulnar nerve shows substantial interindividual variability, limiting the value of single-pattern anatomical descriptions. It remains unclear whether the origins of its motor branches are spatially random or retain reproducible relationships with the neural, anthropometric, and musculotendinous organization of the forearm. This study characterized the normalized spatial distribution of the origins of the main ulnar motor branches supplying the flexor carpi ulnaris (FCU) and the ulnar portion of the flexor digitorum profundus (FDP), and examined their relationships with anatomical reference variables measured during dissection. **Methods**: A cross-sectional cadaveric study was conducted on 16 adult right upper limbs. Fifteen specimens were dissected fresh on the day they were received by the Body Donation Programme; one specimen was frozen at −80 °C, thawed overnight, and dissected the following morning. Standardized anatomical dissection identified 54 main motor branches arising from the ulnar nerve and confirmed their target muscles by direct anatomical continuity (36 FCU and 18 FDP branches). For quantitative mapping, only the origin of each branch on the parent ulnar nerve was recorded using a laser-guided Cartesian coordinate system. Anthropometric dimensions and topographical landmarks were normalized to the corresponding forearm dimensions. Spatial organization was characterized using centroids, 95% data ellipses, Euclidean centroid distances, and a specimen-level cluster bootstrap. Generalized estimating equations (GEE) were used for cluster-aware group comparisons and exploratory multivariable association analysis, while ROC analysis and leave-one-cadaver-out cross-validation quantified internal separation and stability within the cadaveric dataset. **Results**: FCU and FDP branch origins occupied reproducible but partially overlapping normalized spatial territories. Their centroids differed in location, and specimen-level bootstrap resampling supported the stability of the observed separation. Cluster-aware analyses confirmed differences in the proximodistal branch-origin coordinate and in the tendon-crossing landmark. In the multivariable GEE model, the normalized tendon-crossing landmark showed the strongest adjusted association with group membership, while the proximodistal coordinate also remained associated. **Conclusions**: Despite marked interindividual variation in ulnar nerve branching, the origins of its main motor branches exhibit reproducible population-level spatial organization. Their distributions are related to the anatomically confirmed target muscle and to normalized neural, anthropometric, and musculotendinous landmarks measured during dissection. This integrated reference framework supports interpretation of peripheral motor innervation as an organized spatial system rather than a collection of isolated variants. The findings provide an anatomical basis for future imaging and image-guided research; however, the exploratory regression and ROC analyses do not constitute a clinically validated diagnostic model.

## 1. Introduction

Accurate knowledge of peripheral nerve anatomy is essential for diagnostic localization, preoperative planning, and image-guided procedures in the upper limb. High-resolution ultrasonography permits dynamic assessment of peripheral nerves in relation to adjacent muscles, tendons, vessels, and fascial planes, but interpretation depends on anatomical reference models that adequately represent normal variation [1,2,3,4]. Conventional atlases commonly depict a single representative branching pattern and therefore provide only a limited account of the configurations encountered among individuals.

The flexor carpi ulnaris (FCU) and the ulnar portion of the flexor digitorum profundus (FDP) are clinically relevant targets because of their roles in wrist flexion, ulnar deviation, and finger flexion. Their motor branches may be assessed or approached during selective neurectomy, diagnostic motor blockade, peripheral nerve reconstruction, and ultrasound-guided chemodenervation for focal spasticity [5,6,7,8,9]. Accurate localization is particularly important when selective treatment is intended to reduce pathological flexor activity while preserving residual voluntary function.

Cadaveric studies have described the number, origin, trajectory, length, diameter, branching pattern, and muscular distribution of ulnar motor branches [5,10,11,12]. These investigations consistently demonstrate substantial interindividual variation, and comparable variability has been reported for motor branches of the median nerve [13]. This descriptive evidence is indispensable, but it does not establish whether the points at which branches arise from the parent nerve are spatially random or whether they retain organized relationships with the anatomical territories of the muscles they supply.

Most previous investigations have relied on absolute measurements from osseous or surface landmarks. Such values are influenced by forearm dimensions and may combine true biological variability with differences in specimen size. Anthropometric normalization and spatial analysis provide a complementary strategy by representing branch-origin coordinates and anatomical landmarks within a common reference framework [14,15,16,17,18,19,20,21,22]. This permits branch origins to be analysed as population distributions and related to proximal and distal neural landmarks and to the points at which the FCU and FDP tendons cross the interstyloid line.

To date, no study has quantitatively examined whether the normalized spatial distribution of the main ulnar motor branch origins is related to the integrated neural and musculotendinous topography of the forearm. It therefore remains unclear whether anatomically variable branch origins occupy reproducible muscle-specific territories and whether these territories show measurable relationships with the course of the parent nerve and the distal topography of the corresponding tendons.

The aim of this study was to determine whether the origins of the main motor branches arising from the ulnar nerve and supplying the FCU and the ulnar portion of the FDP exhibit reproducible normalized spatial organization and measurable relationships with osseous, neural, anthropometric, and musculotendinous landmarks recorded during dissection. We hypothesized that, despite variation in individual branching morphology, branch origins would form muscle-specific spatial patterns associated with the integrated topography of the forearm.

## 2. Materials and Methods

### 2.1. Study Design

This observational cross-sectional cadaveric study was designed to characterize the anatomical and normalized spatial organization of the points at which the main motor branches supplying the FCU and the ulnar portion of the FDP arose from the ulnar nerve. The study combined standardized anatomical dissection, Cartesian coordinate acquisition, anthropometric measurements, neural reference measurements, tendon-landmark measurements, and quantitative spatial analysis.

Each main motor branch was identified at its origin from the ulnar nerve and followed anatomically until its entry into the target muscle, allowing the target muscle to be established directly by dissection. For quantitative spatial mapping, only the branch-origin point on the parent nerve was registered; neither branch length nor the distance between branch origin and muscular entry was measured. Anthropometric, neural, and musculotendinous reference variables were recorded separately in the same specimen.

The analysis first characterized branch-origin distributions according to the target muscle confirmed by dissection and then examined their relationships with normalized neural, anthropometric, and musculotendinous landmarks. Multivariable and ROC analyses were used only to quantify the strength and internal stability of these anatomical associations; they were not intended to establish a clinical diagnostic classifier.

The study was approved by the Research Ethics Committee of the Faculty of Health Sciences Blanquerna, Universitat Ramon Llull (CER-FCSB) (Approval No. 2025-09-03) and was conducted in accordance with the ethical principles governing research involving donated human bodies. All anatomical specimens were obtained through the Body Donation Programme of the Faculty of Medicine and Health Sciences, University of Barcelona (Campus Clínic, Barcelona, Spain), in accordance with institutional regulations governing body donation for teaching and scientific research.

### 2.2. Anatomical Specimens

Sixteen right upper limbs from sixteen adult body donors were included in this anatomical study. All specimens were obtained through the Body Donation Programme of the Faculty of Medicine and Health Sciences, University of Barcelona (Campus Clínic, Barcelona, Spain), and were available for complete anatomical dissection.

Fifteen specimens were dissected fresh on the same day they were received by the Body Donation Programme, without undergoing freezing or a previous freeze–thaw cycle. One specimen could not be dissected on the day of receipt and was frozen at −80 °C, thawed overnight, and dissected the following morning. No specimen underwent repeated freeze–thaw cycles.

Only right upper limbs were included as an a priori methodological decision to standardize laterality and minimize an additional potential source of anatomical variability. Because access to donated human specimens is inherently limited, restricting the analysis to a single side was intended to maximize control of potentially confounding sources of variation within the available sample. The final sample size of 16 specimens reflected the availability of eligible human donations through the service during the study period.

Only specimens with an intact anterior compartment of the forearm and preserved ulnar nerve anatomy were considered eligible. Upper limbs presenting previous surgical procedures, traumatic injuries, congenital or acquired deformities, advanced degenerative changes, gross anatomical abnormalities, or tissue deterioration that could interfere with identification of the ulnar nerve or its motor branches were excluded.

Each upper limb represented a single anatomical specimen. Within every specimen, every main motor branch arising from the ulnar nerve and supplying either the flexor carpi ulnaris (FCU) or the ulnar portion of the flexor digitorum profundus (FDP) was identified and considered an individual observational unit for the anatomical analyses.

Donor sex and age were recorded, together with forearm anthropometric measurements, to characterize the study sample and provide the reference dimensions required for normalization of the anatomical variables.

### 2.3. Anatomical Dissection

All dissections were performed by a single investigator (A.M.V.) according to a standardized anatomical protocol to ensure procedural consistency throughout the study.

Before dissection, each specimen was positioned with the shoulder in neutral rotation, the elbow in slight extension, and the forearm fully supinated to reproduce the anatomical position. After removal of the skin, subcutaneous tissue, and antebrachial fascia, the anterior compartment of the forearm was carefully exposed while preserving the integrity of the neurovascular structures.

The ulnar nerve was identified proximally and followed distally throughout its extramuscular course. Every muscular branch supplying the flexor carpi ulnaris (FCU) or the ulnar portion of the flexor digitorum profundus (FDP) was meticulously dissected from its origin on the parent ulnar nerve to its penetration into the target muscle. The anatomical relationships between each motor branch and the surrounding muscles, tendons, vascular structures, and fascial planes were preserved throughout the dissection (Supplementary Material).

Motor branch identity was established anatomically by tracing each branch continuously from its origin on the parent ulnar nerve to its penetration into the FCU or the ulnar portion of the FDP. Thus, classification as a motor branch was based on direct anatomical continuity with muscle tissue rather than on topographical position alone.

Whenever a main motor branch divided before entering the target muscle, the number of terminal branches was recorded. The anatomical third of muscle entry (proximal, middle, or distal) and the muscle surface through which each branch entered (anterior, posterior, medial, or lateral) were also documented. However, the main motor branch rather than its terminal divisions constituted the observational unit for all quantitative spatial analyses.

After complete exposure of all motor branches, the specimens were secured to the coordinate acquisition system without altering their anatomical position, thereby preventing shifting during subsequent topographical measurements.

### 2.4. Anatomical Positioning and Coordinate Acquisition System

**Figure 1:**
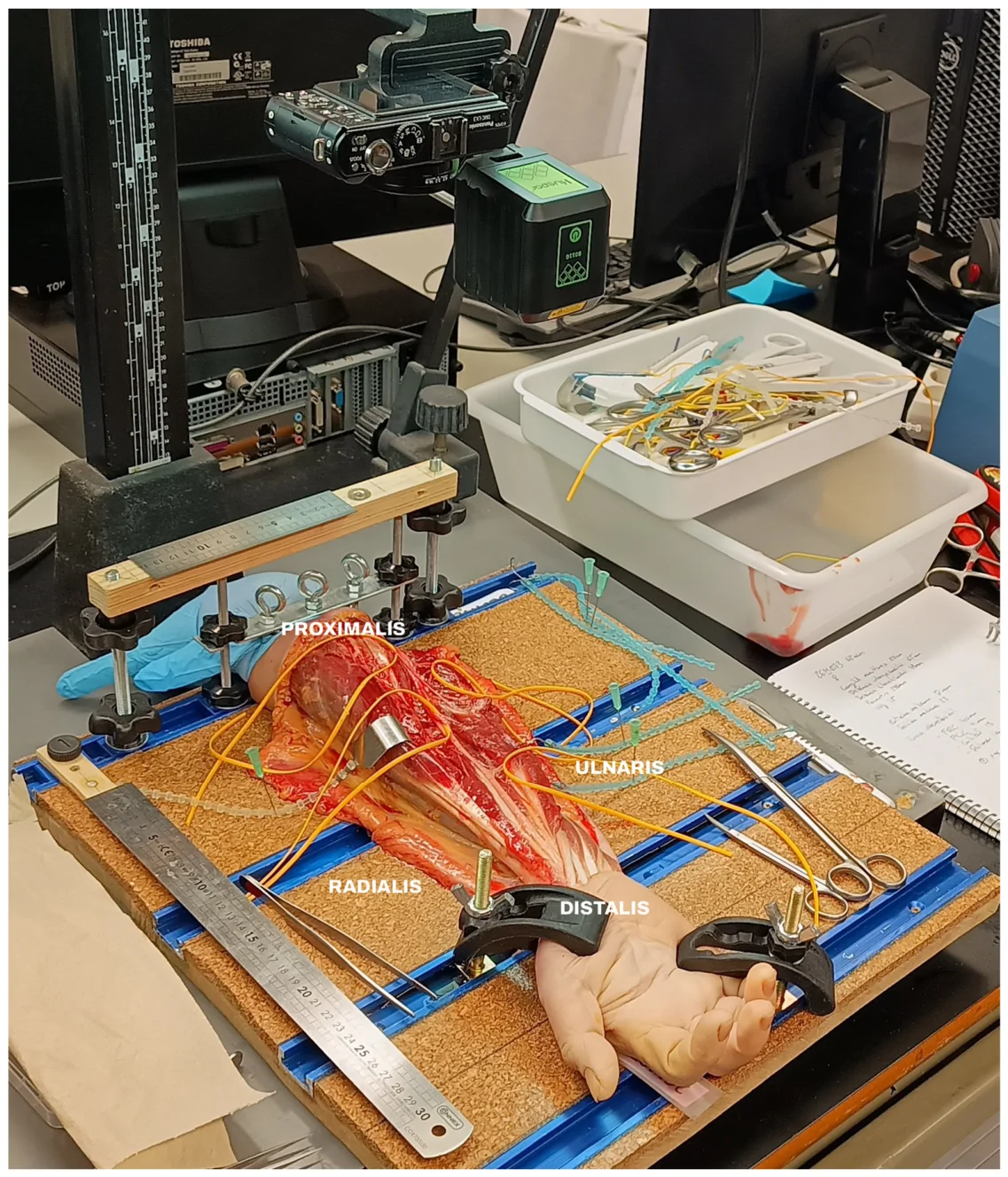
Custom anatomical positioning system used to stabilize the specimen during dissection and coordinate acquisition.

**Figure 2:**
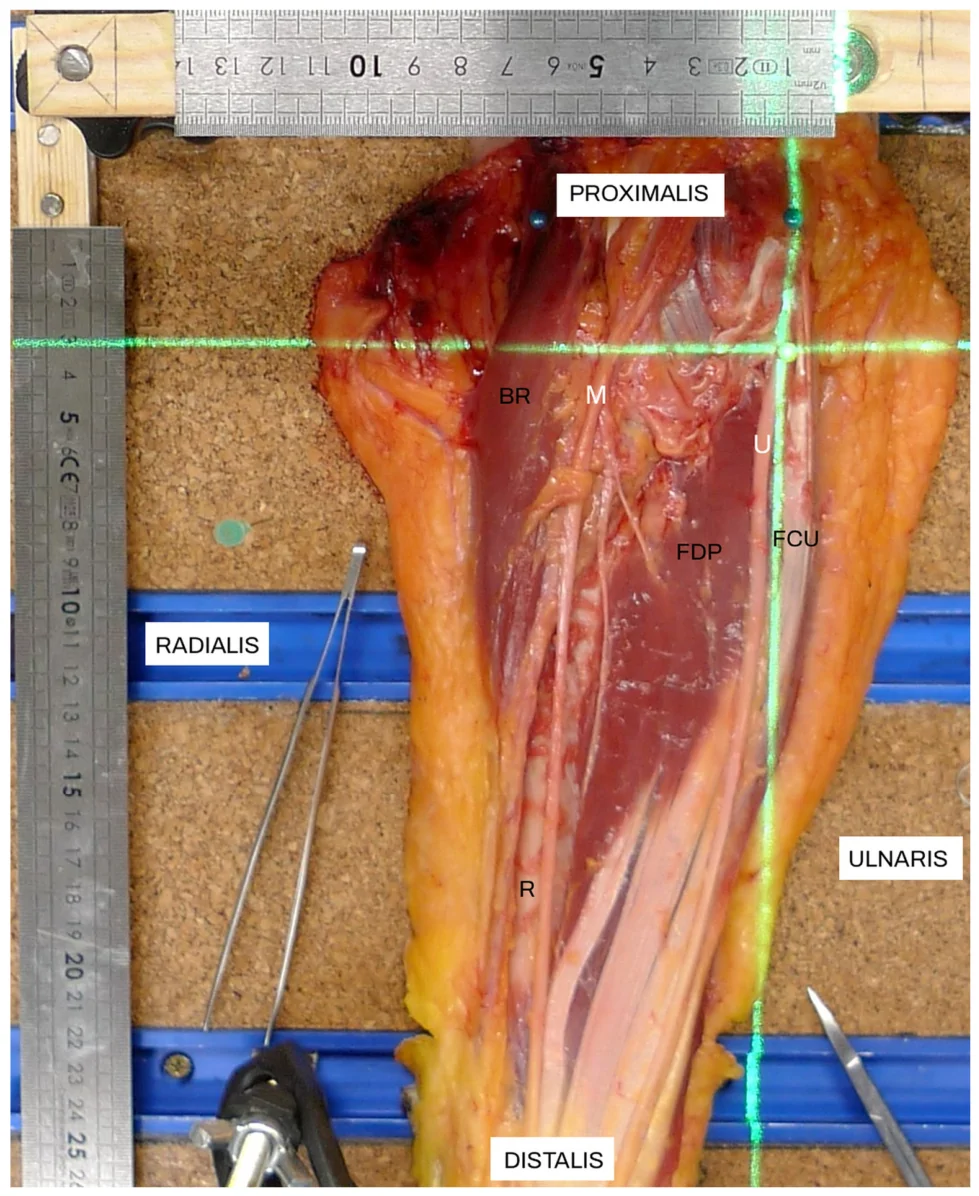
Laser-guided Cartesian coordinate acquisition system used for topographical measurements. R = Radius, BR = Brachioradialis, FCU = Flexor carpi ulnaris, FDP = Flexor digitorum profundus, M = nervus medianus, U = nervus ulnaris.

To ensure reproducible topographical measurements, a dedicated anatomical positioning and coordinate acquisition system was developed for this study (Figure 1 and Figure 2). The system provided standardized positioning of each specimen together with a Cartesian reference framework for the spatial localization of the origins of the main motor branches arising from the ulnar nerve.

Every upper limb was rigidly secured to the positioning platform using two constant osteoarticular reference regions. Proximally, fixation was achieved directly proximal to the interepicondylar line, whereas distally the hand was stabilized immediately distal to the interstyloid line. This dual fixation system prevented movement of the specimen throughout the measurement procedure.

The positioning platform incorporated orthogonal longitudinal and transverse measuring scales aligned with the anatomical axes of the forearm. A laser-guided projection system generated a Cartesian coordinate framework over the dissected specimen, allowing the anatomical origin of each main motor branch to be localized by recording its mediolateral (X) and proximodistal (Y) coordinates relative to the standardized reference system.

The same positioning protocol and reference framework were applied to all specimens to ensure measurement reproducibility and enable subsequent anthropometric normalization of the recorded anatomical variables.

### 2.5. Anatomical Data Acquisition and Normalization

Following complete anatomical dissection, all main motor branches arising from the ulnar nerve and supplying either the FCU or the ulnar portion of the FDP were identified. The target muscle of each branch was confirmed by following the branch from its origin on the parent ulnar nerve to its muscular penetration. For quantitative spatial analysis, only the point at which each main motor branch arose from the ulnar nerve was registered. The number of terminal divisions immediately before muscular penetration, the anatomical third of muscle entry, and the surface of muscular entry were documented descriptively. Branch length and the distance from branch origin to muscular entry were not measured.

A standardized set of anthropometric and topographical reference measurements was obtained during the same dissection procedure. Anthropometric variables included forearm length, interepicondylar distance, interstyloid distance, forearm circumference, and elbow carrying angle. Neural landmarks included the exact point at which the ulnar nerve crossed the interepicondylar line and the point at which it crossed the interstyloid line. Musculotendinous landmarks comprised the exact points at which the FCU and FDP tendons crossed the interstyloid line. All linear landmarks were measured with a caliper in the standardized anatomical position.

The origin of each main motor branch was localized by recording its mediolateral (X) and proximodistal (Y) Cartesian coordinates within the standardized reference framework. The proximal ulnar-nerve crossing was measured along the interepicondylar line, whereas the distal ulnar-nerve passage and the FCU and FDP tendon crossing points were measured along the interstyloid line. These reference measurements were collected systematically for every specimen and were not used to determine branch identity, which had already been established by direct anatomical continuity to the target muscle. They allowed the branch-origin distributions to be examined in relation to osseous, anthropometric, neural, and musculotendinous topography.

To account for interindividual variation in forearm size, all spatial measurements were normalized before statistical analysis. The proximodistal coordinate of each branch origin was expressed relative to forearm length. The mediolateral branch-origin coordinate, the distal position of the ulnar nerve, and the tendon crossing points were expressed relative to interstyloid distance. The proximal crossing position of the ulnar nerve was expressed relative to interepicondylar distance. Each normalized value was calculated by dividing the absolute measurement by the corresponding anatomical reference dimension and multiplying by 100.

The normalized dataset therefore comprised the mediolateral and proximodistal coordinates of each branch origin, the proximal position at which the ulnar nerve crossed the interepicondylar line, its distal position at the interstyloid line, and the FCU and FDP tendon-crossing landmarks at the interstyloid line. These measurements were used to characterize the muscle-specific branch-origin distributions and to explore their relationships with the integrated neural and musculotendinous topography of the forearm.

### 2.6. Measurement Quality Control

All anatomical dissections and identification of the ulnar nerve motor branches were performed by A.M.V. Following dissection and standardized positioning, A.M.V. and A.C.R. independently obtained all quantitative measurements using the same anatomical reference framework and protocol. Each observer measured every quantitative variable three times. The arithmetic mean of the three repeated measurements was first calculated separately for each observer, and the final value entered into the analytical dataset was the mean of the two observer-specific means.

The repeated measurements included the Cartesian coordinates of the origins of the main motor branches and all linear anatomical variables used for normalization and subsequent statistical analyses. A formal reliability assessment was performed using the retained raw repeated-measurement data available for five specimens. Technical error of measurement (TEM), relative TEM (rTEM), and the coefficient of reliability (R) were calculated for the retained anthropometric measurements; TEM and R were also calculated for the retained Cartesian branch-origin coordinates. Interobserver reliability was assessed from the two observer-specific means.

This procedure was used to quantify measurement precision and to minimize random measurement error before spatial normalization and statistical modelling. Because the individual repeated measurements had not been retained for the remaining specimens after observer averaging, the formal retrospective reliability assessment was necessarily restricted to the available five-specimen subset.

### 2.7. Statistical Analysis

Statistical analyses were performed using R version 4.6.0 (R Foundation for Statistical Computing, Vienna, Austria). Each main motor branch remained the anatomical observational unit, but inferential analyses explicitly accounted for clustering of multiple branches within the same cadaver. Statistical significance was established at *p* < 0.05, and all tests were two-sided.

Continuous variables are presented as mean ± standard deviation (SD), whereas categorical variables are presented as absolute frequencies and percentages. Because multiple branches originated from the same cadaver, between-muscle comparisons were reanalysed using generalized estimating equations (GEE) with cadaver identifier as the clustering variable, an exchangeable working correlation structure, and robust standard errors. Cliff’s delta (δ) was retained as a descriptive effect-size measure independent of statistical significance.

The spatial organization of the origins of the main motor branches was investigated using normalized Cartesian coordinates. Geometric centroids were calculated separately for branches supplying the FCU and FDP, and 95% data ellipses were constructed to characterize the spatial dispersion of each group. Ellipse areas, mean distances from individual branch origins to their corresponding centroids, and Euclidean distances between centroids were computed as descriptive measures. The stability of centroid separation was reassessed using a specimen-level cluster bootstrap with 1000 iterations. At each iteration, cadavers rather than individual branches were sampled with replacement, and all branches belonging to each selected cadaver were retained together before recalculating the muscle-specific centroids and their Euclidean distance.

A multivariable GEE logistic model was used as an exploratory association analysis to determine which normalized anatomical variables were most strongly related to the branch-origin distributions while accounting for branches nested within cadavers. The model included five predefined normalized variables representing the integrated anatomical framework: the mediolateral and proximodistal branch-origin coordinates, the point at which the ulnar nerve crossed the interepicondylar line, the position of the ulnar nerve at the interstyloid line, and the tendon-crossing landmark for the corresponding FCU or FDP musculotendinous territory. All five variables were entered simultaneously; no data-driven stepwise variable selection was performed. Odds ratios (ORs) with robust 95% confidence intervals were reported. Given the effective sample size of 16 cadavers and the limited number of FDP branches, all multivariable estimates were considered exploratory and potentially susceptible to overfitting [23].

Because the normalized tendon-crossing landmark showed the strongest adjusted association, ROC analysis was used to describe the degree of internal separation between the two anatomically defined branch-origin distributions. The Youden index identified the sample-specific value that maximized sensitivity and specificity. Positive and negative predictive values and overall accuracy are reported solely as descriptive measures of within-sample separation and are not clinically applicable diagnostic performance estimates.

To account for the clustered structure of the dataset, whereby multiple motor branches originated from the same cadaver, stability was assessed using leave-one-cadaver-out cross-validation (LOCO-CV). At each iteration, all branches from one cadaver were excluded from model fitting and subsequently used as the held-out set. Because complete or quasi-complete separation occurred in some training subsets, bias-reduced logistic regression based on adjusted-score estimation was implemented using the brglm2 package to ensure finite parameter estimates and model convergence in every fold. Internal separation obtained from the tendon-landmark model and the multivariable association model was summarized using AUC, sensitivity, specificity, PPV, NPV, and overall accuracy. Differences between ROC curves were assessed using DeLong’s test. These indices were interpreted only as measures of internal anatomical separation.

All statistical analyses were performed in R using the geepack package for generalized estimating equation (GEE) analyses, the pROC package for ROC curve analyses and AUC comparisons, the brglm2 package for bias-reduced logistic regression, and additional custom scripts for bootstrap resampling and leave-one-cadaver-out cross-validation.

## 3. Results

### 3.1. Gross Anatomy and Branching Pattern

A total of 16 right upper limbs from 16 different cadavers were included in the anatomical study. Across these specimens, 54 main motor branches arising from the ulnar nerve were identified. Of these, 36 branches (66.7%) supplied the flexor carpi ulnaris (FCU), whereas 18 branches (33.3%) supplied the flexor digitorum profundus (FDP). The descriptive anatomical features of the identified branches are summarized in Table 1.

The sample comprised 16 adult body donors, including 8 males and 8 females. Mean donor age was 82.2 ± 9.6 years (range, 63–98 years). Mean forearm length was 232.1 ± 27.8 mm, interepicondylar distance was 64.6 ± 6.5 mm, forearm circumference was 210.1 ± 26.1 mm, elbow carrying angle was 10.8 ± 3.6°, and interstyloid distance was 49.0 ± 5.2 mm. Fifteen upper limbs were dissected fresh on the day of receipt, whereas one specimen underwent a single overnight freeze–thaw cycle as described above.

In the five-specimen subset with retained raw repeated measurements, intraobserver TEM for the anthropometric variables ranged from 0.093 to 0.103 mm, with rTEM values of 0.038–0.197% and reliability coefficients (R) of 0.9983 to >0.9999. Interobserver TEM ranged from 0.021 to 0.030 mm, with rTEM values of 0.009–0.044% and R ≥ 0.9999; the maximum absolute difference between observer-specific means was 0.067 mm. For the 18 retained branch-origin coordinate observations, intraobserver TEM was 0.100 mm for both X and Y coordinates, with R > 0.9998. The observer-specific mean coordinates were identical in this retained subset, yielding an interobserver TEM of 0.000 mm and R = 1.000.

Most main motor branches entered their target muscle within the proximal third of the forearm (79.6%), whereas only 16.7% and 3.7% entered within the middle and distal thirds, respectively. This distribution was similar for branches supplying both the FCU and FDP, although no FDP branch entered the distal third of the forearm.

The site of muscle penetration differed between the two target muscles. Branches supplying the FCU entered predominantly through the lateral surface, whereas those supplying the FDP entered mainly through the medial and anterior surfaces. Posterior entry was uncommon and was observed solely in branches supplying the FCU.

Each main motor branch gave rise to a limited number of terminal branches before penetrating the target muscle. The mean number of terminal branches was 1.22 ± 0.49 (range, 1–3) for branches supplying the FCU and 1.33 ± 0.59 (range, 1–3) for those supplying the FDP.

### 3.2. Spatial Distribution of the Origins of the Main Motor Branches

**Figure 3:**
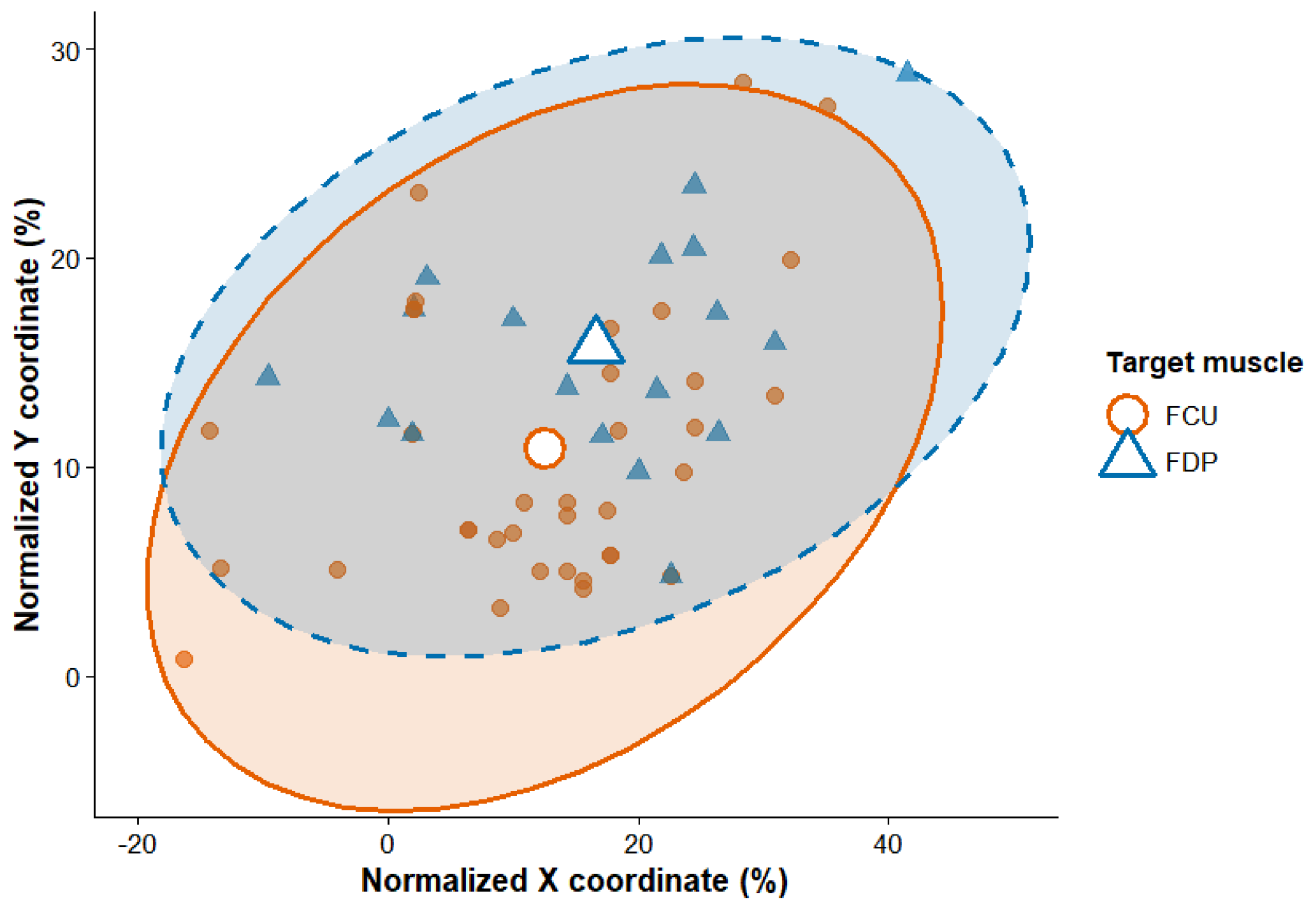
Normalized spatial distribution of the origins of the main ulnar motor branches supplying the flexor carpi ulnaris (FCU) and the ulnar portion of the flexor digitorum profundus (FDP). Each point represents the origin of one anatomically identified branch on the parent ulnar nerve. Group centroids summarize the mean normalized location, and the 95% data ellipses represent the dispersion of each muscle-specific distribution. Partial overlap indicates normal interindividual variability rather than absence of preferential organization.

The spatial distribution of the origins of the main motor branches supplying the flexor carpi ulnaris (FCU) and flexor digitorum profundus (FDP) is shown in Figure 3. The origins of branches supplying both muscles were distributed over a broad region of the normalized coordinate system, with partial overlap between the two distributions.

The centroid of the FCU branches was located at normalized coordinates X = 12.5 and Y = 10.9, whereas the centroid of the FDP branches was located at X = 16.6 and Y = 15.7. Accordingly, the average origin of branches supplying the FDP was positioned more distally and slightly more laterally than that of branches supplying the FCU. The Euclidean distance between the two centroids was 6.30 normalized units.

The spatial dispersion of branch origins was comparable between muscles. The area enclosed by the 95% data ellipse measured 1480.2 normalized units^2^ for the FCU and 1273.6 normalized units^2^ for the FDP. Similarly, the mean distance from individual branch origins to their respective centroids was 11.8 normalized units for the FCU and 12.0 normalized units for the FDP.

Specimen-level cluster bootstrap resampling (1000 iterations) yielded a mean centroid distance of 6.59 normalized units (median, 6.30; 95% percentile interval, 3.43–10.95). The observed centroid distance of 6.30 normalized units was contained within this interval, supporting the stability of the spatial separation while preserving the within-cadaver clustering structure.

Coordinates are expressed within the standardized Cartesian framework. Negative X values indicate medial positions and positive values indicate lateral positions; negative Y values indicate proximal positions and positive values indicate distal positions.

### 3.3. Comparison of Normalized Anatomical Characteristics

The normalized anatomical characteristics associated with the FCU and FDP branch-origin distributions are summarized in Table 2. In cluster-aware GEE analyses, no statistically significant between-group difference was observed for the distal position of the ulnar nerve at the interstyloid line (*p* = 0.600) or for the number of terminal branches (*p* = 0.519). The mediolateral branch-origin coordinate showed a small borderline difference after accounting for clustering (mean difference, 3.00 normalized units; 95% CI, 0.01–5.99; *p* = 0.049).

The proximodistal branch-origin coordinate remained significantly different between the muscle-specific distributions after accounting for within-cadaver clustering (mean difference, 4.85 normalized units; 95% CI, 2.99–6.71; *p* < 0.001), with FDP branches showing larger normalized Y-coordinate values. The tendon-crossing landmarks also differed markedly between the FCU and FDP musculotendinous territories (mean difference, 26.45 normalized units; 95% CI, 21.26–31.65; *p* < 0.001). This remained the largest observed effect (Cliff’s δ = −0.93), with the sign reflecting the ordering of FCU relative to FDP.

### 3.4. Multivariable Analysis of Anatomical Associations

The cluster-aware multivariable GEE model examined the relationship between the anatomically confirmed muscle-specific branch-origin distributions and the five predefined normalized anatomical variables while treating cadaver identifier as the clustering variable.

The normalized tendon-crossing landmark remained the strongest adjusted association with the anatomically defined muscle group (OR = 1.33 per normalized unit, 95% CI: 1.06–1.67; *p* = 0.013). The proximodistal branch-origin coordinate also remained independently associated after adjustment (OR = 1.19, 95% CI: 1.02–1.39; *p* = 0.028). The mediolateral coordinate (OR = 0.97, 95% CI: 0.90–1.05; *p* = 0.472), proximal ulnar-nerve crossing (OR = 1.24, 95% CI: 0.44–3.48; *p* = 0.687), and distal ulnar-nerve position (OR = 1.00, 95% CI: 0.86–1.16; *p* = 0.985) were not independently associated.

Within the study sample, predictions from the complete cluster-aware model produced an AUC of 0.971 and an overall accuracy of 98.1%. These values describe internal anatomical separation only and should not be interpreted as estimates of clinical diagnostic performance.

Because only 16 cadavers contributed data and five predictors were included, the apparent discrimination of the multivariable model must be interpreted cautiously. The effective sample size is governed by the clustered specimen structure rather than by the total number of branches, and model complexity relative to this effective sample size may produce optimistic estimates despite cluster-aware inference and specimen-level internal validation.

### 3.5. Internal Separation and Stability of the Tendon-Related Anatomical Association

**Figure 4:**
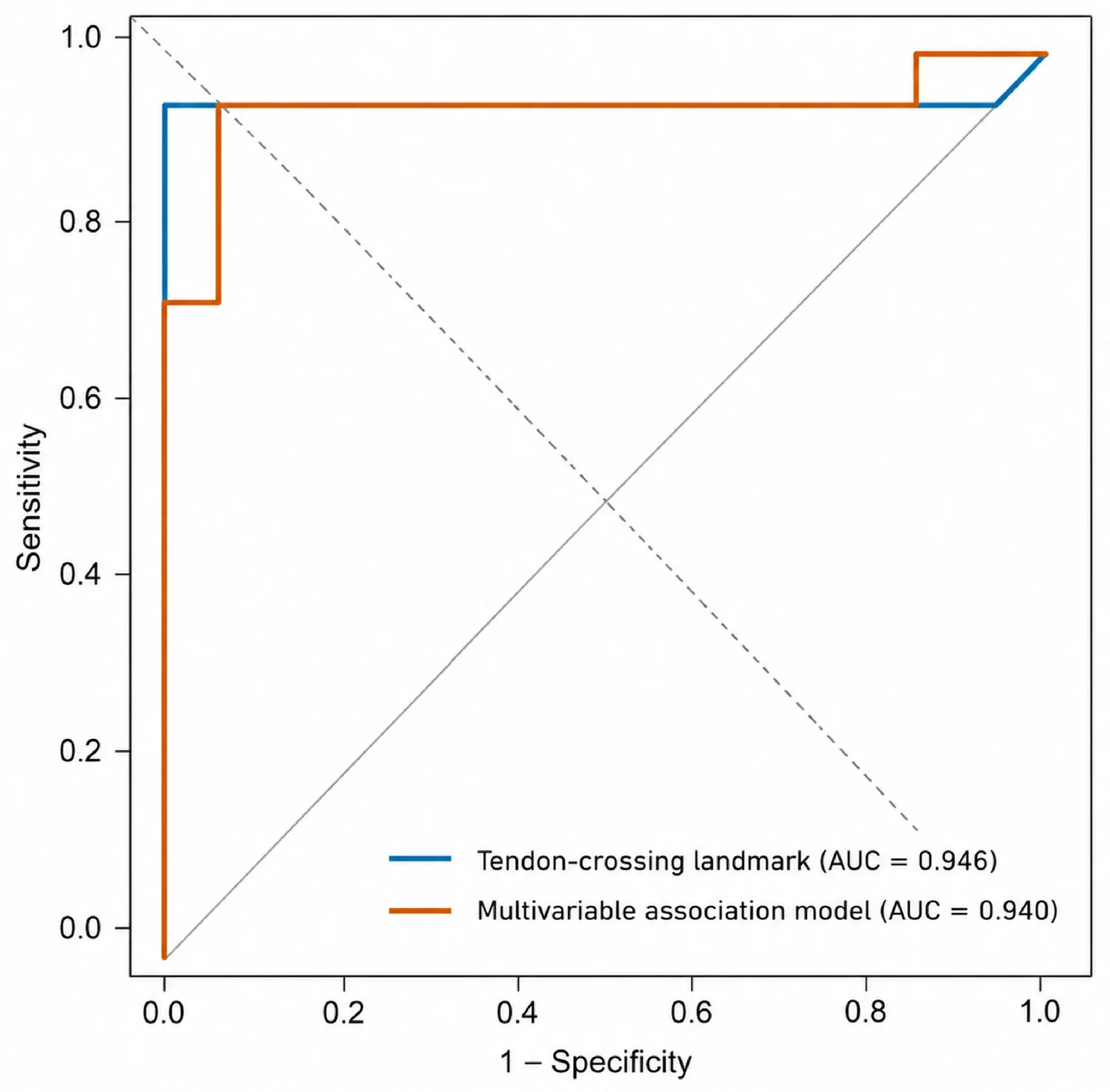
Receiver operating characteristic curves describing internal separation of the anatomically defined FCU and FDP branch-origin distributions using normalized tendon-crossing landmark and the full multivariable association model after leave-one-cadaver-out analysis. The curves summarize within-sample anatomical separation; they do not represent clinical diagnostic validation.

ROC analysis quantified the degree to which normalized tendon-crossing landmark separated the FCU and FDP anatomical distributions in the present sample (Figure 4). The AUC was 0.966 (95% CI, 0.899–1.000). The Youden index identified a sample-specific value of 26.31% of the interstyloid distance. At this value, sensitivity was 94.4%, specificity 100%, positive predictive value 100%, negative predictive value 97.3%, and overall accuracy 98.1% (Table 3). These values describe separation between groups whose target muscles had already been established by dissection and are not proposed as a rule for identifying an unknown branch in clinical practice.

To evaluate the stability of this finding while accounting for the clustered structure of the data, leave-one-cadaver-out cross-validation (LOCO-CV) was performed, with all branches from one cadaver withheld together at each iteration. Following this procedure, the normalized tendon-crossing landmark maintained substantial internal separation, with an AUC of 0.946 (95% CI, 0.840–1.000), a sensitivity of 88.9%, a specificity of 100%, and an overall accuracy of 96.3% (Table 3; Figure 4).

Internal separation obtained from the normalized tendon-crossing landmark was then compared with that of the multivariable association model incorporating all normalized anatomical variables. Following LOCO-CV, the multivariable model achieved an AUC of 0.940 (95% CI, 0.845–1.000), with a sensitivity of 88.9%, a specificity of 94.4%, and an overall accuracy of 92.6% (Table 3; Figure 4). Comparison of the ROC curves showed no significant difference between the two approaches (DeLong test, *p* = 0.630), indicating comparable internal separation within this cadaveric dataset.

## 4. Discussion

### 4.1. Principal Findings and Anatomical Interpretation

This study provides, to our knowledge, the first quantitative assessment of the normalized spatial organization of the origins of the main ulnar motor branches supplying the FCU and the ulnar portion of the FDP in relation to proximal and distal neural landmarks, anthropometric dimensions, and distal musculotendinous landmarks. Previous studies established substantial variability in branch number, origin, trajectory, and muscular distribution [5,10,11,12,13]. The present findings show that this variability does not imply spatial randomness: after normalization for forearm size, branch origins occupied preferential, partially overlapping territories associated with the two target muscles and characterized by distinct centroids.

The overlap between the FCU and FDP territories is anatomically plausible because the muscles occupy adjacent regions, share fascial relationships, and receive branches from the same parent nerve [24,25,26,27]. Complete separation would therefore be biologically unexpected.The relevant observation is that the two distributions retained distinct preferential locations, and bootstrap resampling supported the stability of their centroid separation. Population-level organization can therefore coexist with substantial individual variation.

The strongest adjusted relationship involved the points at which the corresponding tendons crossed the interstyloid line. These landmarks were recorded during the same dissection as topographical characteristics of the FCU and FDP musculotendinous units. Branch identity was established independently by direct anatomical continuity to the target muscle and was not inferred from tendon position; therefore, the analysis is not circular in a strict classificatory sense. Nevertheless, the FCU and FDP variables refer to two anatomically distinct tendons with inherently different mediolateral positions. Consequently, part of the very high discriminatory performance of the tendon-related landmark may reflect the expected spatial separation between the two target tendons themselves, in addition to any broader relationship between branch-origin territory and musculotendinous architecture. The high AUC should therefore be interpreted as an exploratory within-sample anatomical association rather than as evidence that a tendon measurement can identify an unknown motor branch clinically.

Developmental mechanisms offer a plausible, although untested, explanation for this organization. Motor axon guidance and branch stabilization are regulated processes that connect motor axons with their target tissues [28,29,30]. These mechanisms could permit variation in individual trajectories while preserving broader spatial relationships between motor branches and musculotendinous territories. Because development was not examined directly, this interpretation remains a biological hypothesis.

### 4.2. Quantitative Modelling and Internal Validation

The principal methodological contribution is an integrated anatomical reference framework in which branch origins are analysed as spatial populations and related to landmarks measured at different levels of the same anatomical system. These landmarks included the proximal crossing of the ulnar nerve at the interepicondylar line, its distal position at the interstyloid line, the branch-origin coordinates, and the distal crossing points of the FCU and FDP tendons. Population-based approaches are established in computational anatomy and medical imaging because they incorporate normal variability rather than reducing anatomy to a single deterministic pattern [14,15,16,20,21,22]. Applied to peripheral nerve anatomy, this framework complements descriptive dissection by quantifying preferential location, dispersion, overlap, and uncertainty.

Anthropometric normalization was central because absolute coordinates and landmark positions are influenced by specimen dimensions. Longitudinal branch-origin coordinates were expressed relative to forearm length, proximal neural position relative to interepicondylar distance, and transverse distal measurements relative to interstyloid distance [18,19]. The analysis therefore addressed whether proportional anatomical relationships were conserved across specimens rather than whether identical absolute distances occurred in every forearm.

Cluster-aware GEE modelling, specimen-level bootstrap resampling, and leave-one-cadaver-out analysis were used to respect the hierarchical structure of multiple branches nested within cadavers. Resampling cadavers rather than individual branches preserved within-specimen dependence during the bootstrap analysis, while GEE provided robust inference for the group comparisons and multivariable association model. Persistence of separation after omitting each cadaver suggests that the findings were not determined by a single specimen. Nevertheless, the analyses remain exploratory: only 16 cadavers contributed data, the FDP group contained 18 branches, and five predictors were examined simultaneously. These features limit the effective sample size and increase the possibility of overfitting, so odds ratios, ROC-derived measures, AUC, and accuracy should be interpreted cautiously as internally derived anatomical findings rather than externally validated performance estimates [23,31,32].

### 4.3. Diagnostic and Image-Guided Implications

The translational value of the study lies in providing a quantitative anatomical reference framework that can be tested in future imaging studies. High-resolution ultrasonography evaluates peripheral nerves in relation to adjacent tendons, muscles, vessels, and fascial planes [1,2,3,4]. A population-level map of branch-origin territories combined with normalized musculotendinous landmarks may assist the design of standardized scanning protocols and define regions for focused exploration when small motor branches are difficult to visualize directly.

The tendon-related findings are potentially relevant because tendons are generally recognizable during dissection and ultrasonography [1,2]. However, the present study demonstrates an anatomical association, not a validated imaging rule. The sample-specific ROC value was derived from cadaveric measurements in groups whose target muscles had already been established by dissection. Direct correlation with ultrasonography or magnetic resonance neurography is required before any tendon-referenced localization strategy can be proposed for clinical use.

Potential areas for subsequent investigation include selective neurectomy, motor blockade, and image-guided chemodenervation [5,6,7,8,9,33,34]. The present work concerns the origins of the main motor branches on the ulnar nerve and their relationships with target-muscle and musculotendinous topography. It complements studies of complete branch trajectories, motor entry points, and intramuscular arborization, but does not demonstrate improved procedural accuracy, safety, therapeutic response, or patient outcomes.

More broadly, the framework could be adapted to other peripheral nerves and may contribute to probabilistic anatomical atlases that combine three-dimensional anatomy, imaging, and population-level variability [14,15,16,20,21]. Such applications remain prospective and require external anatomical replication, direct imaging correlation, and evidence that the quantitative framework adds value beyond conventional landmarks.

### 4.4. Strengths, Limitations, and Future Research

The principal strengths are the predominant use of fresh human specimens (15 of 16 upper limbs), standardized positioning and coordinate acquisition, systematic anatomical dissection, repeated independent measurements by two observers, anthropometric normalization, spatial statistics, cluster-aware inference, specimen-level bootstrap assessment, and specimen-level cross-validation. The branch-origin coordinates, proximal and distal positions of the parent ulnar nerve, and distal tendon landmarks were measured during the same dissection procedure, allowing their topographical relationships to be analysed within a common normalized framework.

Several limitations should be considered. The study included 16 right upper limbs and 54 motor branches; therefore, the estimated spatial territories and association measures may be sample-specific, and the clustered nature of multiple branches from the same cadaver means that the effective inferential sample size is substantially closer to the number of specimens than to the number of branches. The sample size reflected the availability of eligible donated human specimens through the service. Right-sided specimens were selected deliberately to control laterality as an additional source of anatomical variability, but this design prevents assessment of bilateral symmetry and side-related variation and limits generalization to the left upper limb. Formal measurement reliability could only be calculated retrospectively in the five-specimen subset for which raw repeated measurements had been retained, although agreement in that subset was extremely high. The cadaveric setting does not reproduce tissue tension, dynamic movement, or imaging conditions in living participants. Quantitative analysis was restricted to branch-origin points and did not measure complete extramuscular trajectories, branch length, distance to muscular entry, branch diameter, fascicular architecture, nerve mobility, branching angle, or intramuscular arborization [6,33,34]. Finally, despite the use of GEE and specimen-level validation, the multivariable model remains ambitious relative to 16 cadavers and may be susceptible to overfitting; the tendon-landmark discrimination may also partly encode the intrinsic spatial separation of the FCU and FDP tendons. Independent anatomical and in vivo validation are required.

Future studies should replicate the spatial analysis in larger, multicentre, and demographically diverse anatomical samples; include paired limbs where possible; and directly coregister branch coordinates and tendon landmarks with high-resolution ultrasonography or magnetic resonance neurography. Clinical translation will require testing whether the integrated neural and musculotendinous framework improves interobserver localization, procedural planning, or targeting beyond conventional anatomical landmarks.

## 5. Conclusions

The origins of the main motor branches of the ulnar nerve supplying the FCU and the ulnar portion of the FDP exhibited reproducible normalized spatial organization despite substantial individual variation. They formed preferential, partially overlapping territories, differed along the proximodistal axis, and showed a strong association with the topography of the corresponding tendon crossing points at the interstyloid line. The measured proximal and distal positions of the parent ulnar nerve showed weaker relationships.

These findings support an integrated, population-level interpretation of peripheral motor anatomy in which branch-origin coordinates, neural landmarks, anthropometric dimensions, and musculotendinous structures are components of a common spatial system. The study provides a transferable quantitative anatomical reference framework for future peripheral nerve imaging and image-guided research. The cluster-aware regression and ROC results describe exploratory internal anatomical associations and do not constitute a clinically validated diagnostic model.

## Figures and Tables

**Table 1:** Descriptive anatomical features of the main motor branches arising from the ulnar nerve.

Feature	Overall (n = 54)	FCU (n = 36)	FDP (n = 18)
Main motor branches, n (%)	54 (100)	36 (66.7)	18 (33.3)
Third of muscle entry, n (%)			
Proximal	43 (79.6)	29 (80.6)	14 (77.8)
Middle	9 (16.7)	5 (13.9)	4 (22.2)
Distal	2 (3.7)	2 (5.6)	0 (0)
Surface of muscle entry, n (%)			
Lateral	29 (53.7)	28 (77.8)	1 (5.6)
Medial	13 (24.1)	4 (11.1)	9 (50.0)
Anterior	10 (18.5)	2 (5.6)	8 (44.4)
Posterior	2 (3.7)	2 (5.6)	0 (0)
Terminal branches, mean ± SD (range)	—	1.22 ± 0.49 (1–3)	1.33 ± 0.59 (1–3)

Abbreviations: FCU, flexor carpi ulnaris; FDP, flexor digitorum profundus; SD, standard deviation.

**Table 2:** Comparison of normalized anatomical variables associated with the FCU and FDP branch-origin distributions. *p* values are from generalized estimating equations accounting for clustering of branches within cadavers; Cliff’s δ is retained as a descriptive effect-size measure.

Variable	FCU (n = 36)	FDP (n = 18)	Cliff’s δ	*p* Value
Ulnar nerve position (% interstyloid distance)	17.97 ± 7.88	19.19 ± 9.10	−0.05	0.600
Mediolateral coordinate (% interstyloid distance)	12.52 ± 12.46	16.60 ± 12.97	−0.18	0.049
Proximodistal coordinate (% forearm length)	10.93 ± 6.80	15.73 ± 5.53	−0.44	<0.001
Tendon-crossing landmark (% interstyloid distance)	14.09 ± 5.69	42.66 ± 11.41	−0.93	<0.001
Terminal branches (n)	1.22 ± 0.48	1.33 ± 0.59	−0.09	0.519

**Table 3:** Internal separation obtained from the normalized tendon-crossing landmark before and after leave-one-cadaver-out analysis and comparison with the full multivariable association model.

Metric	Initial Tendon-Landmark Model	LOCO Tendon-Landmark Model	LOCO Multivariable Association Model
AUC (95% CI)	0.966 (0.899–1.000)	0.946 (0.840–1.000)	0.940 (0.845–1.000)
Sensitivity (%)	94.4	88.9	88.9
Specificity (%)	100.0	100.0	94.4
Positive predictive value (%)	100.0	100.0	88.9
Negative predictive value (%)	97.3	94.7	94.4
Accuracy (%)	98.1	96.3	92.6

## Data Availability

The data presented in this study are available from the corresponding author upon reasonable request. The data are not publicly available because they form part of an ongoing line of anatomical research.

## Supplementary Materials

The following supporting information can be downloaded at: https://drive.google.com/file/d/1pgx34_vKxGv1Eh7m2aPZCit7_gdX24yA/view?usp=sharing (accessed on 28 August 2026).

## Abbreviations

Abbreviation	Definition
AIC	Akaike information criterion
AUC	Area under the receiver operating characteristic curve
CI	Confidence interval
FCU	Flexor carpi ulnaris
FDP	Flexor digitorum profundus
LOCO-CV	Leave-one-cadaver-out cross-validation
NPV	Negative predictive value
OR	Odds ratio
PPV	Positive predictive value
ROC	Receiver operating characteristic
SD	Standard deviation
